# Impact of the host response and osteoblast lineage cells on periodontal disease

**DOI:** 10.3389/fimmu.2022.998244

**Published:** 2022-10-11

**Authors:** Mi Zhou, Dana T. Graves

**Affiliations:** ^1^ Department of Stomatology, Union Hospital, Tongji Medical College, Huazhong University of Science and Technology, Wuhan, China; ^2^ School of Stomatology, Tongji Medical College, Huazhong University of Science and Technology, Wuhan, China; ^3^ Hubei Province Key Laboratory of Oral and Maxillofacial Development and Regeneration, Wuhan, China; ^4^ Department of Periodontics, School of Dental Medicine, University of Pennsylvania, Philadelphia, PA, United States

**Keywords:** bone remodeling, periodontal ligament, gingiva, growth factor, lymphocyte, osseous, osteogenic, stromal

## Abstract

Periodontitis involves the loss of connective tissue attachment and alveolar bone. Single cell RNA-seq experiments have provided new insight into how resident cells and infiltrating immune cells function in response to bacterial challenge in periodontal tissues. Periodontal disease is induced by a combined innate and adaptive immune response to bacterial dysbiosis that is initiated by resident cells including epithelial cells and fibroblasts, which recruit immune cells. Chemokines and cytokines stimulate recruitment of osteoclast precursors and osteoclastogenesis in response to TNF, IL-1β, IL-6, IL-17, RANKL and other factors. Inflammation also suppresses coupled bone formation to limit repair of osteolytic lesions. Bone lining cells, osteocytes and periodontal ligament cells play a key role in both processes. The periodontal ligament contains cells that exhibit similarities to tendon cells, osteoblast-lineage cells and mesenchymal stem cells. Bone lining cells consisting of mesenchymal stem cells, osteoprogenitors and osteoblasts are influenced by osteocytes and stimulate formation of osteoclast precursors through MCSF and RANKL, which directly induce osteoclastogenesis. Following bone resorption, factors are released from resorbed bone matrix and by osteoclasts and osteal macrophages that recruit osteoblast precursors to the resorbed bone surface. Osteoblast differentiation and coupled bone formation are regulated by multiple signaling pathways including Wnt, Notch, FGF, IGF-1, BMP, and Hedgehog pathways. Diabetes, cigarette smoking and aging enhance the pathologic processes to increase bone resorption and inhibit coupled bone formation to accelerate bone loss. Other bone pathologies such as rheumatoid arthritis, post-menopausal osteoporosis and bone unloading/disuse also affect osteoblast lineage cells and participate in formation of osteolytic lesions by promoting bone resorption and inhibiting coupled bone formation. Thus, periodontitis involves the activation of an inflammatory response that involves a large number of cells to stimulate bone resorption and limit osseous repair processes.

## Introduction

There are two major forms of periodontal disease, gingivitis and periodontitis. Gingivitis, inflammation of the gingiva, always precedes periodontitis but does not necessarily lead to it. In gingivitis, damage to the gingival tissue is reversible. Periodontitis is characterized by loss of connective tissue attachment to the teeth and loss of bone that surrounds the tooth, which is generally thought to be permanent. Both gingivitis and periodontitis involve inflammation from both the innate and adaptive immune responses. The immune responses affect bone remodeling through impact on osteoblast lineage cells, periodontal ligament fibroblasts, and osteoclasts, which impact bone resorption and bone coupling. Recent single cell analysis has shed light on the types of cells involved including fibroblasts, epithelial cells, vascular cells and leukocytes. However, the role of bone-associated and periodontal ligament cells is less well studied. This review aims to describe how inflammation generated by the innate and adaptive immune response affects osteoblast lineage cells and the contribution of the latter to bone resorption and uncoupled bone formation. The cellular interactions are critical in understanding the formation of osteolytic lesions that are characteristic of periodontitis.

## Osteoblasts, osteocytes, periodontal ligament and osteoclast cells

**Figure 1:**
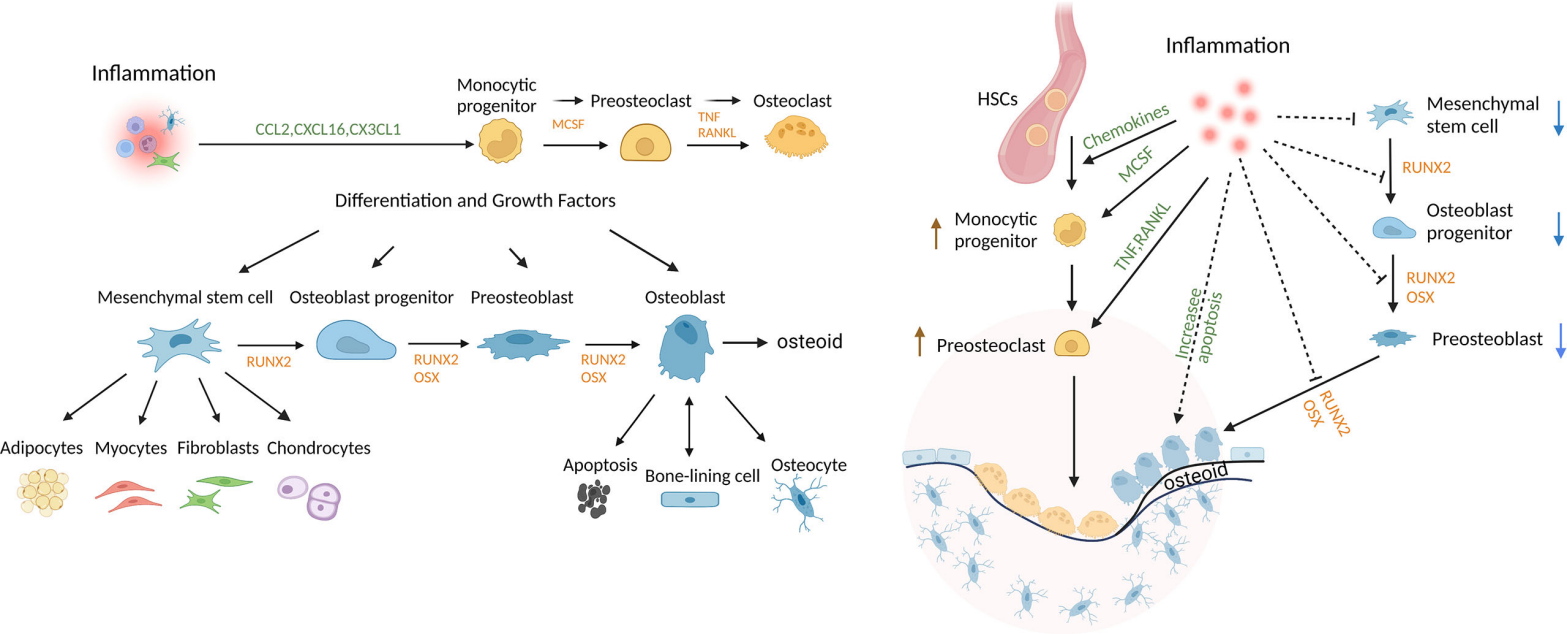
Regulation of bone and the impact of inflammation. Mesenchymal stem cells (MSCs) can differentiate into multiple lineages and are regulated by growth and differentiation factors that induce transcriptional programs that are lineage-specific. Osteoclasts are of myeloid lineage. Inflammation promotes osteoclastogenesis, in part, by inducing osteocytes to produce chemokines, inflammatory cytokines and RANKL. Inflammation also reduces coupled bone formation by inducing osteoblasts/osteocytes to produce inhibitors of Wnt signaling, DKK2 and sclerostin, interfering with differentiation at several steps, limiting the production of bone matrix, osteoid, and inducing apoptosis of mesenchymal stem cells, OB progenitors, OB and osteocytes. Thus, bone lining cells, OB and osteocytes play an important role in maintaining periodontal bone mass through secreting factors that regulate the balance of osteoclastogenesis and osteogenesis.

Osteoblast lineage cells are formed from the differentiation of mesenchymal precursors to osteoblasts. The osteoblast lineage cells play a critical role in bone development, growth, and maintenance. Bone undergoes remodeling in vertebrates throughout life. Progenitors differentiate to osteoblasts through the activity of transcription factors such as runt-related transcription factor 2 (Runx2) and osterix (Osx) (1, 2). Mature matrix-producing osteoblasts synthesize collagen-rich unmineralized matrix, osteoid. Osteoblasts have 3 fates; they can form bone-ling cells, osteocytes, or undergo apoptosis (1, 2). Bone-lining cells cover bone surfaces and exhibit properties of immature mesenchymal cells with multi-lineage potential. These cells proliferate as an early step in bone repair (3, 4). Osteocytes, which are incorporated into the osteoid matrix, are the most abundant osteoblast lineage cells and account for approximately 95% of cells in mature bone tissue (5, 6). Osteocytes play important roles in bone remodeling. The differentiation of osteoblast lineage cells and osteoclast cells is shown in **Figure 1**. Osteocytes can limit bone formation by production of sclerostin (6, 7) and can stimulate bone formation by production of growth factors such as insulin-like growth factor (IGF)-1 or the chemokine CCL5 that recruits osteoblasts to sites of resorption and promotes bone coupling (6, 8, 9). Bone lining and other cells produce macrophage colony-stimulating factor (M-CSF) to induce formation of osteoclast progenitors, while osteocytes and other cells contribute to bone resorption through production of receptor activator of nuclear factor κ-Β ligand (RANKL). Apoptotic osteocytes promote nearby osteocytes to secrete bone-resorbing factors (6–8). In periodontitis animal models, deletion of RANKL expression specifically in osteocytes inhibits osteoclastogenesis and bone loss, demonstrating that osteocytes are an important source of RANKL in this disease (10, 11).

Cementoblasts are large cuboidal cells that produce cementum. Both cementoblast precursors and osteoblast progenitors are resident cells in the periodontal ligament (PDL). The differentiation of cementoblasts from precursors is less well studied than osteoblasts but is thought to involve many of the same transcriptional programs (12, 13). Recently, two distinct stem cell populations in the periodontal ligament have been identified by scRNA-seq that contribute to cementoblast differentiation (14). Microarray analysis indicates that there are some differences in the expression of regulatory genes that control cementoblasts and osteoblasts (15). The formation of cementoblasts and production of cementum is thought to be a critical step in periodontal regeneration following bone loss due to periodontitis.

PDL fibroblasts are mesenchymal cells with multifunctional properties that participate in various cellular activities and respond to inflammatory stimuli (16, 17). PDL cells exhibit similarities to tendon cells and immature mesenchymal cells with some osteoblastic characteristics as demonstrated by 2.5kb collagen-1α1 promoter reporter activity *in vivo*. Bacterial dysbiosis in mice stimulates expression and activation of nuclear factor kappa-B (NF-κB) in PDL fibroblasts, bone-lining cells and osteocytes, which produce chemokines and RANKL to stimulate bone resorption (18). PDL cells influence immune responses through production of cytokines and chemokines in response to an oral dysbiosis (17, 18). A recent study examined PDL cells by single cell RNA-seq. Two major populations of mesenchymal cells were identified, Scx^+^ (scleraxis) and Mkx^+^ *(*mohawk homeobox), which is expressed in tendon cells (19). Scx^+^ PDL cells are located in the central part of the PDL and produce collagen. Mkx^+^ cells are seen throughout the PDL and produce oxytalan fibers and proteoglycan (19). In addition to differentiating to osteoblasts, PDL cells differentiate to cementoblast-like cells and chondrocytes (20). PDL cells express periostin, which stimulates expression of RUNX2 and differentiation to osteoblasts (21). Interestingly, periostin expression is downregulated by periodontal disease. Periodontal ligament stem cells have been categorized as “high osteogenic” or “low osteogenic” potential (19). Those with low osteogenic potential have hypermethylated DNA and low expression of genes that regulate osteoblast differentiation. Thus, PDL cells with high osteogenic potential have reduced methylation and greater capacity to form osteoblastic cells. Persistent methylation may serve as a brake to limit differentiation of PDL subpopulations (22).

Osteoclasts originate from cells of the mononuclear phagocyte lineage and are responsible for bone resorption (23). Osteoclast precursors can be formed from immature monocytic cells, immature monocyte-derived dendritic cells and resident macrophages (24, 25). Osteoclast precursors are recruited to sites of inflammation by chemokines such as CCL2, CXCL16, and CX3CL1. Some chemokines support the proliferation of precursors or their differentiation to osteoclasts (26). Osteoclasts also produce chemokines CCL3, CCL5, CCL9 and CCL22 to amplify osteoclastogenesis and bone resorption (27, 28). Differentiation of monocytic cells to osteoclast precursors is stimulated by M-CSF and multi-nucleated osteoclast formation is induced *via* RANKL (29). Prostaglandins and a number of cytokines can indirectly promote osteoclastogenesis including TNF, IL-1, IL-6, IL-7, and IL-23 by stimulating production of RANKL. Conversely, there are a number of anti-osteoclastogenic cytokines such as IL-3, IL-4, IL-10, and interferon gamma (IFN-γ) (8, 30). Several co-stimulators are needed to facilitate osteoclastogenesis including osteoclast-associated receptor (OSCAR), immunoreceptor tyrosine-based activation motif (ITAM), DNAX associated protein 12kD size (DAP12) and FcϵR1 gamma chain (FcRγ) (31, 32). Secreted osteoclastogenic factor of activated T cells (SOFAT) is produced by activated T cells and stimulates bone resorption in the absence of osteoblasts or RANKL (33). Dendritic cell-specific transmembrane protein (DC-STAMP) plays an essential role in the formation of multinucleated osteoclasts (34, 35). Knock-down of DC-STAMP abrogates cell–cell fusion. Osteoclasts isolated from DC-STAMP knockout (KO) mice have single nuclei due to deficiency in cell-cell fusion and exhibit mild osteopetrosis (36). Osteoclasts express carbonic anhydride to produce carbonic acid (H_2_CO_3_) and to generate protons that are released into lacunae. This leads to formation of HCl at the resorption site to create an acidic microenvironment with pH of 4, which dissolves hydroxyapatite. Osteoclasts also secrete cathepsin K and MMP-9 to degrade type-1 collagen and other bone matrix proteins (30).

Remodeling activity of alveolar bone occurs at a significantly higher rate than skeletal bone. The high degree of mechanical stress caused by mastication plays a role in the high turnover rate under both physiological and pathological conditions. Mechanical loading enhances the production of RANKL to increase alveolar bone turnover. PDL fibroblasts play an important role in regulating alveolar bone remolding in response to mechanical forces. These cells can produce RANKL, IL-1β and TNF-α to stimulate alveolar bone remodeling (37).

## Stimulation of bone formation

A number of pathways stimulate osteoblasts to promote bone formation. They include Wnt, Hedgehog, bone morphogenetic protein (BMP), transforming growth factor β (TGF-β) and Notch signaling. Wnts are secreted glycoproteins that activate β-catenin, a transcription factor that induces osteoblast differentiation and activity. The canonical Wnt/β-catenin pathway plays a vital role in regulating embryonic development and bone formation (38, 39). There are approximately 20 different Wnt ligands ranging from Wnt-1 to Wnt-16b. Wnts activate intracellular signaling pathways by binding to one of 10 different receptors in the frizzled family. In some cases a co-receptor is required such as lipoprotein receptor-related protein (LRP)-5 or LRP-6. Wnt signaling stimulates proliferation of progenitors and stimulates osteoblast differentiation and survival (40). Inhibitors of Wnt/β-catenin signaling reduce or block bone formation and include Axin2, Sclerostin and Dickkopf-1 (Dkk1). DKK1 competitively binds to the Wnt co-receptor LRP-6 (41).

FGF (fibroblast growth factor) represents a large family of growth factors that play a role in bone formation (42, 43). FGF2, formerly known as basic-FGF, promotes bone formation through increased proliferation of osteoprogenitors, stimulation of angiogenesis and increased osteoblast differentiation. FGF-2 can upregulate BMP2 signaling and increase β-catenin levels in calvarial osteoblast precursors (44). FGF-18 up-regulates RANKL expression in osteoblasts (45). IGF (insulin -like growth factor) consists of IGF-1 and -2. IGF-1 has both local and systemic effects and binds to IGF-1 cell-surface receptors to stimulate proliferation of osteoblast precursors, formation of osteoid and prevent apoptosis, thereby enhancing bone formation and maintaining bone mass (46). IGF-1 is synthesized by cells such as preosteoblasts, mature osteoblasts, osteocytes, and osteoclasts (47, 48). IGF-1 signaling also promotes osteoclast differentiation (49). IGF-2 plays a role in growth during fetal development and is important in the maintenance of stem cell populations (50).

TGF-β consists of more than 30 family members and includes activin, nodal, BMPs, growth and differentiation factors (GDFs) and TGF-β1, TGF-β2 and TGF-β3. TGF-β superfamily members regulate bone cell proliferation, differentiation, and function (51, 52). SMAD proteins are the intracellular effectors of TGF-β signaling (53). TGF-β1 stimulates formation of committed osteoprogenitors *via* the ERK pathway and activation of the transcription factor, Runx2 (54). TGF-β2 is important in embryonic development (55, 56). BMP2 and TGF-β synergistically induce mesenchymal stem cell differentiation to committed osteoblasts (57). TGF-β3 induces endochondral bone formation and recruits endogenous HMSCs to initiate bone regeneration (58). BMP (bone morphogenetic protein) enhances MSC differentiation to osteoblasts while opposing proliferative pathways stimulated by Wnt (59). BMPs are expressed by many cells, including osteocytes and osteoblasts. The best characterized with regard to bone formation are BMP-2 and BMP-7. BMP-2 is released from the bone matrix as a result of bone resorption and contributes to differentiation of osteoblasts by inducing Runx2 (59, 60). Osteoblast lineage commitment is also increased by BMP-7 signaling to promote femurs and tibiae bone formation (61).

Hedgehog proteins that stimulate bone formation consist of sonic hedgehog (Shh) and Indian hedgehog (Ihh). Ihh and Shh stimulate osteoblast differentiation and bone formation (62–64). Ihh inhibits MSC differentiation to adipocytes and promotes differentiation to osteoblasts. Ihh signaling also acts synergistically with the Wnt and BMP pathways to promote bone formation (65). Shh upregulates osterix, an osteoblast-specific transcription factor that induces gene expression, which promotes differentiation of preosteoblasts to mature osteoblasts (66, 67). Shh may also play a role in regenerating the periodontium since it stimulates cementoblast differentiation (68).

The Notch pathway is initiated by interaction between adjacent cells and typically suppress bone formation. There are five Notch ligands in mammals, delta like-1, -3 and -4 and jagged (Jag) -1 and -2, which are transmembrane proteins that bind to notch receptors -1, -2, -3 and -4 (69). Notch-1 or -2 both suppress differentiation of mesenchymal progenitors to osteoblasts (70). Notch suppresses the Wnt pathway in MSCs to inhibit MSC differentiation to osteoblasts. Notch-1 signaling in osteocytes induces S*ost* and *Dkk1* expression to limit bone formation in femurs (71). Notch-1 and -2 can inhibit osteoclastogenesis directly or indirectly by inducing osteoprotegerin (OPG) in osteoblasts and osteocytes. In contrast, Notch-3 induces RANKL in osteoblasts and osteocytes (72).

Several other factors are important in bone formation and/or bone coupling and have been recently reviewed (73). They include platelet derived growth factor (PDGF) that stimulates proliferation of osteoblast precursors and stimulates periodontal bone formation *in vivo* (74). Semaphorins are a large family of extracellular signaling molecules that affect bone. Sema3A and Sema3B help maintain bone mass by suppressing bone resorption and increasing bone formation (75).

### Bone remodeling

Bone remodeling consists of two major processes; existing bone is resorbed by osteoclasts, which initiates a process of new bone formation by osteoblasts (76, 77). This cycle is regulated precisely between osteoblasts and osteoclasts and occurs in 4 steps: activation, resorption, reversal and formation. The remodeling processes involve a basic multicellular unit (BMU) that includes osteoclasts, mononuclear cells that are osteoblast precursors and osteoblasts (76, 77). Activation of remodeling can be stimulated by local factors such as microdamage or inflammation that induces osteocytes to produce factors that stimulate bone resorption (78). A key factor is thought to be the death of osteocytes caused by micro-damage or immune cell stimulation, which induce osteocytes to produce pro-osteoclastogenic factors (68). Remodeling may also be stimulated by local factors or in response to systemic factors such as parathyroid hormone (PTH) (79).

Bone resorption in a BMU in humans lasts approximately 3 weeks and the bone formation phase 3 to 4 months (76, 77). During resorption, osteoclasts release factors from bone matrix such as IGF-1 and TGF-β to recruit and activate osteoblasts. Bone resorption is terminated by osteoclast apoptosis and is followed by reversal. Chemotactic signals released by apoptotic osteoclasts (e.g. CXCL16, CLL5, CLL20, and CLL12) and from bone matrix during resorption (e.g. TGF-β) attract stromal-derived mesenchymal cells to the sites of repair. CCL2 produced by osteoblastic cells is also thought to stimulate recruitment of osteoprogenitors cells (80, 81). These precursor cells cover the resorbed bone surface. They are stimulated to differentiate into osteoblasts by factors such as BMPs and Wnts in both periodontal and iliac bone (5, 82, 83). Bone formation involves the production of type I collagen, proteoglycans, glycosaminoglycans, alkaline phosphatase, osteonectin, osteopontin, osteocalcin and other proteins (84–86). Osteocalcin is the most abundant non-collagenous protein in bone, is expressed by osteoblasts and promotes mineralization by directing the alignment of apatite crystals with collagen fibers. Alkaline phosphatase regulates mineralization in two different ways. It hydrolyzes inorganic pyrophosphate, which is a natural inhibitor of mineralization. Alkaline phosphatase also provides inorganic phosphate that is needed to synthesize hydroxyapatite. Ostonectin promotes mineral deposition and crystal growth. Osteopontin dissipates energy and inhibits microfacture propagation (84–86).

#### Unloading or inactivity

Healthy bone will adapt or remodel in response to stress, which represents a functional adaptation (87). Osteocytes sense disuse inactivity and promote bone resorption by regulating osteoclastogenesis and osteoblasts (68, 88, 89). Inactivity reduces Wnt1 expression and increases sclerostin production, which inhibits the Wnt/β-catenin pathway resulting in decreased osteoblast formation and activity. Unloading promotes osteocyte apoptosis by increased expression of pro-apoptotic genes. Apoptotic osteocytes enhance the expression bone-resorptive cytokines such as RANKL and reduce OPG expression (90–92).

#### Rheumatoid arthritis (RA)

Rheumatoid arthritis is a chronic autoimmune disease that targets joint cartilage and bone to cause disability (93). Autoimmune induction is influenced by genetic, epigenetic and environmental factors such as cigarette smoke and dust exposure (94). A factor that may be an important trigger is the development of auto-antibodies to citrullinated proteins that are formed as a result of peptidyl arginine deiminase enzymes in oral and gut bacteria (94, 95). Single-cell RNA-seq from human synovial tissue has defined cell populations that drive joint inflammation in rheumatoid arthritis. They include complex interactions between synovial fibroblasts, monocytes, autoimmune-associated B-cell, T-helper and T-follicular cells (96). RA upregulates proinflammatory cytokines such as IL-1β, IL-6, and TNF-α expression (97). The inflammatory environment induces and activates MMPs and other enzymes that cause cartilage degradation (98). In addition, RA induces TNF-α, RANKL and IL-17A expression to stimulate bone resorption and factors that inhibit coupled bone formation to create osteolytic lesions (96, 99). Sources of RANKL in RA are osteocytes, synovial fibroblasts, T-cells, B-cells, monocytes and macrophages (97–101). Bone coupling is blocked since inflammatory cytokines that inhibit osteoblast differentiation and matrix production also stimulate osteocytes to produce DKK1 and sclerostin (102–104). B-cells also suppress osteoblast differentiation by production of IL-35 and IL-6 (105–107).

#### Post-menopausal osteoporosis

Osteoporosis is characterized by decreasing bone mass and alteration of bone structure that increases bone fragility and risk of fracture (108). Osteoblasts and osteocytes have estrogen receptors that induce intracellular signaling that promote maintenance of bone mass (109). Estrogen signaling inhibits osteocyte and osteoblast apoptosis to enhance bone formation (109). Estrogen deficiency reduces expression of IGF-1, TGF-β, BMP and Wnt that suppress bone formation (110, 111). Another mechanism through which this occurs is estrogen-reduced bone resorption. Calcitonin production is increased by estrogen, which inhibits osteoclast activity, while estrogen promotes apoptosis of osteoclasts. Estrogen receptor signaling down-regulates RANKL and upregulates OPG in osteocytes to reduce bone resorption (110, 112). Lack of estrogen causes increased RANKL and reduced OPG in MSCs, T-cells and B-cells to promote bone resorption (110, 113). Estrogen deficiency also increases cytokines such as IL-7, IL-15 and IL-17A, thus promoting osteoclastogenesis (114, 115). Like other bone pathologies, post-menopausal osteoporosis affects both bone resorption and coupled bone formation.

## Periodontal disease

A long-standing paradigm is that the subgingival microbiota shifts from a composition that is “normal” to one of “dysbiosis” to induce periodontitis. The specifics of the dysbiosis are largely unknown but are generally thought to involve a decrease in the number of beneficial symbionts and increase in the number of pathobionts. This concept is consistent with the historical perspective of a decrease in gram-positive aerobes and an increase in gram-negative anaerobes, although longitudinal studies are needed to more conclusively establish this paradigm (116, 117). Another possibility is that there is a shift in the inflammatory response so that in periodontitis inflammation is in closer proximity to bone thereby involving osteoblast lineage cells to cause bone uncoupling and net bone loss (118)

As mentioned above, gingivitis and periodontitis involve inflammation from both the innate and adaptive immune responses. Resident cells such as epithelial cells and fibroblasts as well as innate immune cells play a key role in generating an inflammatory response to bacterial challenge. The latter include neutrophils, monocytes, macrophages, eosinophils, basophils, mast cells, dendritic cells, NK cells, γδ T-cells, NKT-cells, and innate lymphoid cells (119, 120). Perturbation by trauma or bacteria stimulate chemokine production that induces innate immune cells to migrate to the site of perturbation. Neutrophils are one of the most common leukocytes in the periodontium and play a critical role in protecting the host from microbial challenge. Neutrophils produce respiratory bursts, releasing a number of factors through degranulation, are phagocytic and produce neutrophil extracellular traps (NET). Interestingly, both extremes of excessive numbers of neutrophils and neutrophil deficiency are linked to severe periodontal disease (121). Neutrophils produce a number of factors that are pro-inflammatory and can stimulate bone resorption or inhibit coupled bone formation. They include IL-1, TNF, IL-6 and other factors (122). Monocytes/macrophages play an important role in both bone resorption and bone formation and have multiple phenotypes that are classically defined as pro-inflammatory (M1) and pro-healing (M2). Recent evidence indicates that macrophages can exhibit simultaneously M1 and M2 phenotypes (123, 124). M1 macrophages initiate osteoclastogenesis and the first stages of osseous repair by stimulation of pro-inflammatory factors such as IL-1, IL-6, IL-12, and TNF-α (123, 125). M2 macrophages are activated by IL-4 and IL-13 (Th2 related cytokines) to resolve inflammation and inhibit osteoclastogenesis. M2 macrophages also release bone morphogenetic protein-2 (BMP-2) to stimulate bone formation and clear apoptotic cells to facilitate bone regeneration (124–126). Osteal macrophages (osteomacs) are a subtype of resident tissue macrophages and are an integral component of bone tissue (127, 128). Osteomacs support bone remodeling by inducing osteoblast differentiation and bone formation.

Dendritic cells are found in low numbers but are the predominant antigen presenting cells (129). Dendritic cells shape the immune response by directing the formation of specific T-helper subsets. They also produce cytokines that affect B-cell activation and activation of innate immune cells (129). Inhibition of dendritic cell function in mice causes a reduced adaptive immune response and increases periodontal disease susceptibility (130).

Adaptive immunity is performed by T-cells that express classic alpha and beta T-cell receptors and B-cells. CD4^+^ T (helper) cells include naive CD4^+^ T cells, T-memory cells and other CD4^+^ Th cells (119, 120, 131). The latter include Th1, Th2, Th9, Th17, Tregs, and T-follicular helper (Tfh). Th1 cells produce IL-1 and IFN-γ and Th17 cells produce IL-17A. Th1 and Th17 cells are associated with enhanced inflammation (119, 120, 131). The production of these cytokines can induce expression of destructive enzymes that promote removal of injured tissue but may also induce tissue destruction and stimulate RANKL expression to degrade bone (132–134). Cytokines produced by Th1 and Th17 cells also induce the production of chemokines to recruit neutrophils and macrophages to enhance the innate immune response (120, 134). Th2 and Treg lymphocytes produce cytokines such as IL-4, IL-10, IL-27, IL-35, and TGF-β that resolve inflammation or reduce bone resorption (135–137). Th2/Treg-secreted cytokines also upregulate OPG to inhibit bone resorption (132, 133). Th9 cells increase T-cell expansion and survival and Tfh cells found in the spleen and lymph nodes enhance antibody production by B-cells (133, 137, 138). CD8^+^ cytotoxic T-lymphocytes (CTL) release vesicles containing perforin and granzyme, inducing death of target cells. CD8^+^ T-regs reduce osteoclastogenesis by producing IL-10 and TGF-β (139).

Antigen primed B-cells are stimulated by Th2 cells to differentiate to plasms cells. B-cell proliferation is stimulated by APRIL and BLyS, which are important for their survival, proliferation, and maturation. The expression of APRIL and BLyS upregulated in gingiva from animals and humans with periodontitis (140). In addition to producing antibodies, B-cells produce pro-inflammatory cytokines and can contribute to tissue destruction (120, 131). The production of opsonizing antibodies makes the targeting of microbes by phagocytic cells more efficient. B-regs are a B-cell subset, producing IL-10, IL-35, and TGF-β1 to reduce inflammation and inhibit bone resorption (139, 141). Mice with B-cell deficiency have increased ligature induced periodontal bone loss compared to wild -type mice, suggesting that B-cells may be protective (142). Thus, in periodontitis the production of pro-inflammatory cytokines outweighs the protective effect of cytokines such as IL-4, IL-10 and TGF-β. Interestingly, humoral immunity becomes less effective with age as reflected by a reduced capacity to generate an antibody response (143). This occurs in an environment in aging of increased cellular senescence and production of pro-inflammatory cytokines such as IL-1, IL-6 and TNF, a process referred to as “inflammaging” (143). It is possible that these factors converge to increase susceptibility to periodontitis with age.

Periodontitis involves the formation of an osteolytic lesion in which there is both bone resorption and suppression of coupled bone formation. Activation of the immune response plays an important role in reducing coupled bone formation, which leads to increased net bone loss due to diminished repair of the osteolytic lesion. In animal models, oral dysbiosis induces inflammation in bone lining cells and osteocytes as reflected by increased nuclear localization of NF-κB in these cells (10). In mice, when NF-kB activation is blocked in osteoblastic cells but not other cell types, dysbiosis-induced periodontal bone loss is inhibited (10). This is due to two distinct mechanisms. Blocking NF-kB activation reduces RANKL expression by osteocytes and other osteoblastic cells to diminish bone resorption. In addition, NF-kB in osteoblast lineage cells inhibits coupled bone formation. NF-kB impairs bone formation by inhibiting differentiation of osteoblast precursors, indirectly stimulating apoptosis of osteoblastic cells or their precursors and reducing production of bone osteoid (104, 144). The latter occurs because NF-kB inhibits the expression of proteins that make up osteoid (104). Bacterial dysbiosis significantly increases the number of TNF-α producing cells and increases bone-lining cell death 10-fold. The increased apoptosis is functionally significant since treatment with an apoptosis-specific inhibitor reduces periodontal bone loss through increased coupled bone formation. When the adaptive immune response is stimulated by oral bacteria, coupled bone formation is further inhibited (145). Thus, activation of NF-kB in osteoblast precursors, osteoblasts and osteoclast precursors plays a key role in periodontitis by promoting bone resorption and limiting coupled bone formation.

**Figure 2:**
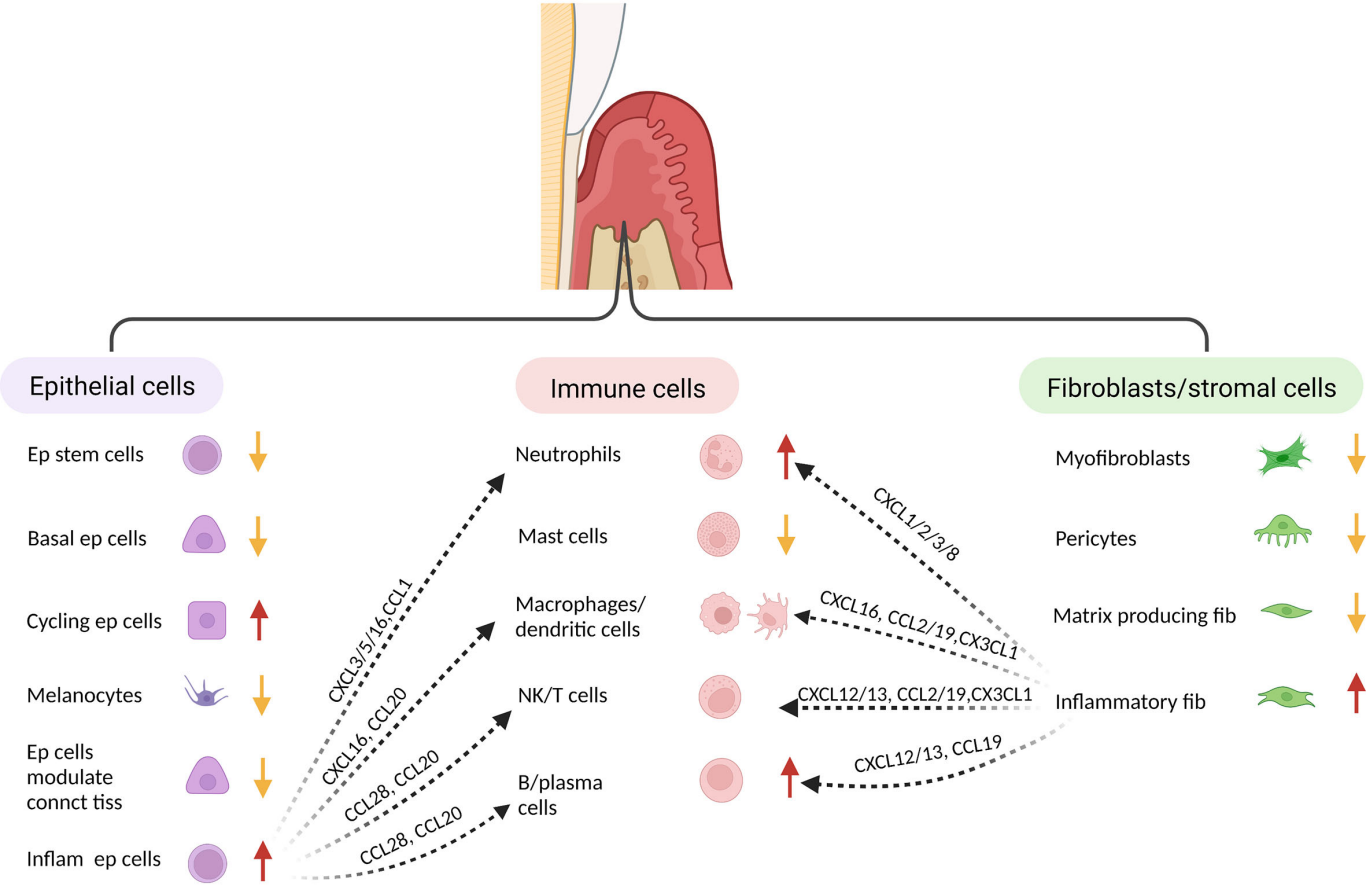
Single cell analysis indicates identifies distinct fibroblastic/stromal, epithelial and immune cell sub-populations. When subjected to transcriptional analysis, several distinct clusters can be identified within the major groups of cells isolated from healthy and inflamed gingiva, suggesting specialized function. When compared to healthy tissue, gingiva associated with periodontal inflammation exhibits a decrease in several epithelial populations (but an increase in inflammatory epithelial cells, an increase in neutrophils and B-cells, and a decrease in fibroblasts/stromal cells (but an increase in inflammatory fibroblasts). The various cell populations from each major group respond to challenge and interact to generate an inflammatory host response. This table was adapted from information in ref: (138) and (140).

Single cell analysis has provided new insight into periodontal disease. In one study epithelial and fibroblastic stromal cell populations were identified as key cells that produce antimicrobial factors or chemokines that stimulate neutrophil recruitment and other leukocyte subsets (145) (**Figure 2**). In health, stromal cells produce neutrophil chemoattractants that may contribute to maintaining homeostasis, whereas in periodontitis there is a shift to intercellular signals produced by macrophages, mast cells, T-cells, and B-cells (145–147). In periodontitis there is a loss of stromal cell populations, particularly myofibroblasts and pericytes. Periodontitis is associated with an expansion of B-cells, which may contribute to an overall increase in inflammation through antigen presentation and cytokine production (147). Periodontitis is linked to an increase in TNF and IL-1, which has previously been shown to play a key role in periodontal bone loss (118, 148). Periodontal disease reduces the number of myeloid derived suppressor cells compared with healthy controls, which could potentially contribute to greater inflammation (146). Single cell RNA-seq also suggests that Ephrin-Eph receptor signaling is more abundant in healthy periodontal tissue than in periodontal disease tissue (146). This may be significant since Ephrin-Eph signaling is important in maintaining angiogenesis and proliferation of immature mesenchymal cells. Ephrin ligand-eph receptor signaling appears to also occur between endothelial cells and pre-osteoblasts. Thus, endothelial cells may play an important role in maintaining the number of MSCs in health (146). These studies point to the complex interactions between various cell types and the role that resident cells such as epithelial cells and fibroblasts have in providing protective signaling that maintains homeostasis.

An important issue that has not been firmly established is the spatial location of inflammation as it pertains to gingivitis and periodontitis. New techniques may provide data in the future which helps distinguish inflammatory events in gingivitis and periodontitis. We have proposed, based on cellular changes in experimental periodontitis in non-human primates, that the location of inflammation in relationship to bone is a key factor (145). This concept is hypothetically supported by findings above that the impact of inflammation on bone-lining cells and osteocytes may play a critical role in the repair of osteolytic lesions (11).

### Systemic conditions and periodontitis

Bone coupling is an essential component of periodontitis and is significantly affected by the impact of inflammation on osteoblast differentiation and activity. Three conditions that are known to have a significant impact on bone are diabetes, smoking and aging. These three systemic conditions are linked to reduced bone coupling resulting in net bone loss and will be briefly reviewed here.

### Diabetes and periodontitis

Diabetes increases the risk of periodontitis approximately 2 fold and increases its severity compared to non-diabetics (149, 150). The impact of diabetes is inversely proportional to the level of glycemic control. Both type I diabetes (T1D) and T2D increase inflammatory events in the periodontium, due to a number of factors including high levels of glucose, reactive oxygen species (ROS) and advanced glycation end-products, each of which increases activation of NF-κB and cytokine expression such as TNF-α or IL-17A (151). Multiple cell types in periodontal tissues are affected by diabetes including leukocytes, vascular cells, MSCs, periodontal ligament fibroblasts, osteoblasts, and osteocytes.

Increased alveolar bone resorption in human diabetics with periodontitis is linked to an increased RANKL/OPG ratio (151–153). Diabetes increases the intensity and duration of an inflammatory infiltrate and osteoclastogenesis in experimental periodontitis (154). Periodontal inflammation is prolonged in both type 1 and type 2 diabetic mice and in humans in response to bacterial challenge (155, 156). Macrophages in gingiva from periodontitis induced by ligature placement in rats have higher expression of the NF-κB consistent with a hyper-inflammatory environment (157). Functional studies show that a TNF inhibitor significantly reduces expression of other cytokines, diminishes leukocyte infiltration and bone resorption in diabetic rats, indicating that cytokine dyregulation is an essential component of diabetes-enhanced periodontal bone loss *in vivo* (158). Diabetes-enhanced inflammation has a dramatic effect on gene expression in the gingiva as shown by mRNA profiling in animals treated with a TNF inhibitor (159). Diabetes both up- and down-regulates genes in a TNF-dependent manner. It predominantly up-regulates genes involved in the host response, apoptosis, and coagulation/homeostasis/complement and down-regulates mRNA levels of genes that regulate metabolism. Moreover, the anti-inflammatory transcription factor, PPAR-α is up-regulated during the resolution of periodontal inflammation in normal animals and suppressed by diabetes. This may contribute to prolonged gingival inflammation in diabetics (159). Expression of inflammatory mediators is also upregulated in the diabetic periodontium in osteocytes and PDL fibroblasts (11). The expression of RANKL by osteocytes and PDL cells functionally plays a significant role in the higher levels of bone loss seen in diabetic animals *in vivo* (11). The increased production may be due to the impact of high glucose levels as high glucose increases NF-κB transcriptional activity (18).

Diabetes also leads to greater bone loss by reducing coupled bone formation. The amount of new alveolar bone formation following an episode of periodontal bone resorption is almost 3-fold higher in normoglycemic compared to diabetic animals (153). Diabetes interferes with the new bone formation by reducing osteoblast differentiation and matrix production, which can be linked to diminished expression of transcription factors needed for bone formation *in vivo* including Runx-2, Dlx5 and c-fos (159). Some of these events can be directly linked to increased activation of NF-kB by diabetes. In addition, sclerostin release by osteocytes is synergistically increased by elevated levels of advanced glycation end products, increased levels of ROS and TNF in the diabetic periodontium, which can reduce bone formation by inhibiting the Wnt pathway (160, 161). Other mechanisms by which diabetes may impair coupled bone formation is through high glucose-suppressed IGF-1 expression (162), reduced MSC proliferation due to the impact of advanced glycation end products and inhibition of osteoblast differentiation by inflammatory mediators and oxidative stress (152, 163, 164). In addition, *in vivo* experiments demonstrate that high levels of TNF in diabetic animals suppress proliferation of bone-lining cells due to the impact of reduced growth factor expression including FGF-2, TGFβ-1, BMP-2, and BMP-6 (157).

An unexpected finding in an animal model was that diabetes-enhanced inflammation modified the oral microbiota to render it more pathogenic (165). This was demonstrated at two levels. The dysbiosis induced by diabetes could be partially reversed by inhibiting inflammation in the diabetic gingiva by treatment with an IL-17 antibody (166). Thus, the development of diabetes created a change in the microbial composition that was in part, dependent upon the level of inflammation. In addition, the transfer of bacteria from diabetic donors to germ-free hosts induced more bone loss than transfer of bacteria from normoglycemic animals. Studies in humans are consistent with animal studies and indicate that diabetic subjects have lower oral bacterial diversity and an increase in bacterial taxa that are associated with pathogenicity (166–168). Interestingly, two other systemic diseases associated with greater levels of systemic inflammation, rheumatoid arthritis (RA) and lupus erythematosus have increased susceptibility to periodontal diseases and alterations in oral bacterial taxa associated with periodontal disease (167).

### The impact of smoking on periodontal disease

Smoking significantly increases the incidence and progression of periodontitis and is proportional to the amount of exposure (169, 170). Although the effects of smoking lingers, outcomes improve with the length of time of smoking cessation (171). Tobacco smoke contains over 4000 potential chemicals including nicotine (172). Tobacco smoke clearly enhances the risk of periodontitis but its mechanisms have not been firmly established. However, there are several plausible mechanisms that may occur concurrently.

Cigarette smoke increases bone resorption, which may be due to a number of factors One involves the direct effect of smoke components on osteoclasts. Cigarette smoke exposure increases alveolar bone remodeling and osteoclastogenesis (173). The increase in osteoclasts can be explained mechanistically due to smoke-induced down-regulation of the caspase 3 pathway in these cells. In addition, cigarette smoke affects osteoclast-precursors so that they are predisposed to form osteoclasts (173). Long term smokers have increased oxidative stress that enhances osteoclast formation and survival. This may be due to increased production of ROS and reduced expression of antioxidants (174). Smoking may also promote bone resorption by increasing the RANKL/OPG ratio (172, 175, 176). However, the effect of tobacco smoke on cytokine levels has been inconclusive (171, 177, 178).

Another mechanism that may come into play is the impact of tobacco smoking on down-regulating the reparative capacity of fibroblasts, periodontal ligament cells, osteoblasts and cementoblasts to a bacteria/inflammation stimulated injury of periodontal tissues (179). Periodontal ligament fibroblasts (PDLFs) display reduced cell viability, proliferation and migration with increasing concentrations of cigarette smoke extract (180). Smoking promotes osteoblasts apoptosis and may cause uncoupling to increase bone loss. Exposure to smoke in mice decreases the number of osteoblast progenitors and reduces osteoblast differentiation (181, 182). In addition, sclerostin and DKK1 are upregulated in periodontitis patients with smoking, both of which inhibit bone formation by negatively impacting the Wnt pathway (183). This finding is consistent with observations that smoking reduces bone formation in fracture healing (184, 185). Thus, smoking may reduce repair of gingiva, PDL and bone, creating greater loss of attachment and reduced bone coupling to enhance net bone loss.

Smoking may also alter the bacterial composition. Smokers with periodontitis have increased bacterial anaerobes compared to non-smokers (186). They include anaerobic bacteria such as *Fusobacterium, Treponema, P. gingivalis, Tannerella forsythia* and other pathogenic bacteria (167). The shift in bacteria may contribute to increased risk and severity of periodontitis. In contrast to tobacco-based cigarettes, e-cigarettes appear to be less pathogenic in promoting periodontal disease (185). However, e-cigarettes are not harmless as they increase the representation of pathogens in the oral microbiota and increase proinflammatory signals (187).

Taken as a whole, there is strong epidemiologic evidence that smoking negatively impacts periodontitis. Although there is no clear mechanism there are several that are plausible including increased bone resorption due to the impact of smoke on osteoclasts, decreased repair capacity including coupled bone formation, changes in the effectiveness of the immune response and increased oxidative stress and microbial changes that increase pathogenicity.

### The effect of aging on periodontal disease

Periodontitis and osteoporosis are linked to both inflammation and aging. Although aging is not a direct cause of periodontitis, aging can affect the periodontal environment to potentially affect bone resorption and bone coupling. Enhanced cytokine production that stimulates osteoclastogenesis and inhibits osteoblastic bone formation are increased with aging. The increased tendency toward inflammation may be due to increased levels of oxidative stress, reduced antioxidants, cellular senescence and accumulation of advanced glycation end products (188). Osteoporosis and periodontitis are both associated with bone resorption. NF-κB related cytokines play a central role in periodontitis and is increased with aging, which may be linked to increased inflammation associated with aging. As discussed above, increased activation of NF-kB in osteoblasts lineage cells increases RANKL expression and inhibits bone formation (188). Aging reduces gingival fibroblast proliferation and migration, fiber density, organic matrix production, and cellular mitotic activity in the periodontal ligament. Meanwhile, aging increases mRNA levels of MMP-2, MMP-8, which promote extracellular matrix degradation (189).

Aging may affect periodontal tissues by altering the host response. There is reduced effectiveness of the adaptive immune response with aging (189). Aged mice have reduced recruitment of dendritic cells in response to bacterial challenge, which may be due to reduced DC migration caused by high glucose levels or high levels of advanced glycation end products (190). Reduced DC activation of the adaptive immune response could contribute to an increased susceptibility to bacterial challenge. This is consistent with observations that P. gingivalis-induced dysbiosis has a greater impact on aged versus young mice (190). While aged mice appear to have a reduced adaptive immune response to bacterial challenge, aging has been shown to increase the innate immune response to P. gingivalis. Old mice have increased periodontal bone loss with higher levels of IL-1β, TNF, TLR2 and complement C5a receptors (191, 192). Reduced adaptive immunity but increased innate immunity may increase the risk and severity of periodontal disease. Aging also impacts the oral microbiota. A positive correlation between age and the presence of *Fusobacterium, P. gingivalis, F. alocis, Pasteurellaceae*, and *Prevotella* has been reported (192). It has been suggested that increased inflammation due to factors such as TNF, causes a shift in the oral microbiota that facilitates bacterial dissemination, which in turn may accelerate aging processes (193).

## Conclusion

Periodontal disease is thought to be induced by a combined innate and adaptive immune response to a bacterial dysbiosis that affects the gingiva. Inflammation is initiated by resident cells including epithelial cells and fibroblasts, which recruit immune cells. The enhanced inflammatory state triggers the expression of cytokines that induce osteoclastogenesis and bone resorption. Most importantly, inflammation leads to periodontitis by interfering with the proliferation of osteoblast progenitors, inhibiting osteoblast differentiation and reducing the production of osteoid matrix inhibiting repair of an osteolytic lesion. Similarly, systemic conditions that affect local inflammation in the periodontium may influence bone resorption and bone coupling, leading to greater periodontitis. The inflammation may also induce an amplification loop in which the bacterial composition becomes more pathogenic because of changes in substrate availability linked to the inflammatory state of the gingival tissue. Thus, all of the cells present in the periodontal tissues are likely to participate one way or the other in the development of gingival inflammation that can transition to periodontitis and loss of supporting bone for the teeth. New advances in in single cell RNA-seq and spatial transcriptomics along with enhanced bioinformatic analysis is likely to shed new light on these processes. The greater sharing of databases amongst researchers is a positive development that should further accelerate the process of discovery.

## Author contributions

DG conceived the manuscript. MZ and DG wrote the manuscript and created the figures. DG reviewed and edited the manuscript. Both authors contributed to the article and approved the submitted version.

## Funding

Funding for this manuscript was provided to DG from the National Institute of Dental and Craniofacial Research, DE02192110 and DE01773212.

## Conflict of interest

The authors declare that the research was conducted in the absence of any commercial or financial relationships that could be construed as a potential conflict of interest.

## Publisher’s note

All claims expressed in this article are solely those of the authors and do not necessarily represent those of their affiliated organizations, or those of the publisher, the editors and the reviewers. Any product that may be evaluated in this article, or claim that may be made by its manufacturer, is not guaranteed or endorsed by the publisher.
